# Supplementary material for: Widespread nociceptive maps in the human neonatal somatosensory cortex
Source: eLife. 2022 Apr 22;11:e71655. doi: 10.7554/eLife.71655 (PMC9090328; doi:10.7554/eLife.71655)
Supplement: Transparent reporting form [file elife-71655-transrepform1.docx]

***eLife’s* transparent reporting form**

We encourage authors to provide detailed information *within their submission* to facilitate the interpretation and replication of experiments. Authors can upload supporting documentation to indicate the use of appropriate reporting guidelines for health-related research (see [EQUATOR Network](http://www.equator-network.org/%20)), life science research (see the [BioSharing Information Resource](https://biosharing.org/)), or the [ARRIVE guidelines](http://www.plosbiology.org/article/info:doi/10.1371/journal.pbio.1000412) for reporting work involving animal research. Where applicable, authors should refer to any relevant reporting standards documents in this form.

If you have any questions, please consult our Journal Policies and/or contact us: [editorial@elifesciences.org](mailto:editorial@elifesciences.org).

**Sample-size estimation**

- You should state whether an appropriate sample size was computed when the study was being designed
- You should state the statistical method of sample size computation and any required assumptions
- If no explicit power analysis was used, you should describe how you decided what sample (replicate) size (number) to use

Please outline where this information can be found within the submission (e.g., sections or figure legends), or explain why this information doesn’t apply to your submission:

Sample sizes in this study were calculated based on previous published data in high impact papers where single trial noxious stimulation or multiple mechanical stimulations have yielded significant results with group sample sizes of 5-15 (Bartocci et al.,2006) and 10-15 (Arichi et al., 2012), respectively

**Replicates**

- You should report how often each experiment was performed
- You should include a definition of biological versus technical replication
- The data obtained should be provided and sufficient information should be provided to indicate the number of independent biological and/or technical replicates
- If you encountered any outliers, you should describe how these were handled
- Criteria for exclusion/inclusion of data should be clearly stated
- High-throughput sequence data should be uploaded before submission, with a private link for reviewers provided (these are available from both GEO and ArrayExpress)

Please outline where this information can be found within the submission (e.g., sections or figure legends), or explain why this information doesn’t apply to your submission:

This is not applicable to our work as we do not use replicates. However, exclusion/inclusion criteria for participants and trials can be found in the ‘Participants’, ‘Innocuous mechanical stimulation’, and ‘Data pre-processing’ sections of “Materials and Methods’.

**Statistical reporting**

- Statistical analysis methods should be described and justified
- Raw data should be presented in figures whenever informative to do so (typically when N per group is less than 10)
- For each experiment, you should identify the statistical tests used, exact values of N, definitions of center, methods of multiple test correction, and dispersion and precision measures (e.g., mean, median, SD, SEM, confidence intervals; and, for the major substantive results, a measure of effect size (e.g., Pearson's r, Cohen's d)
- Report exact p-values wherever possible alongside the summary statistics and 95% confidence intervals. These should be reported for all key questions and not only when the p-value is less than 0.05.

Please outline where this information can be found within the submission (e.g., sections or figure legends), or explain why this information doesn’t apply to your submission:

Statistical analysis methods can be found in the ‘Channel-wise data analysis’ and ‘Data analysis in image space’ sections of ‘Materials and Methods’

Pre-processed data for every fNIRS channel and stimulus type can be seen in Figure 1, which has a source data table (source data 1) that provides the peak amplitude and p-values.

Due to the ‘noisy’ nature of raw fNIRS signals, these would not be informative to include. However raw data has been uploaded to an online repository (‘Data sharing’ section of Materials and Methods).

Results in Figure 3 and Figure 3 – figure supplement 1, show null distributions, 95% CIs, and exact p-values. P values and mean values are reported again in Figure 3 – Source Data and in the ‘Results’ section.

(For large datasets, or papers with a very large number of statistical tests, you may upload a single table file with tests, Ns, etc., with reference to sections in the manuscript.)

**Group allocation**

- Indicate how samples were allocated into experimental groups (in the case of clinical studies, please specify allocation to treatment method); if randomization was used, please also state if restricted randomization was applied
- Indicate if masking was used during group allocation, data collection and/or data analysis

Please outline where this information can be found within the submission (e.g., sections or figure legends), or explain why this information doesn’t apply to your submission:

This does not apply to our submission as infants were allocated to groups opportunistically. 1) Infants were recruited to the lance stimulus group due to the clinical need for a blood test. 2) Although some infants had both hand and foot stimulations, others were limited to one due to 1) location of cannulas, pulse oximeters etc. and 2) becoming unsettled

**Additional data files (“source data”)**

- We encourage you to upload relevant additional data files, such as numerical data that are represented as a graph in a figure, or as a summary table
- Where provided, these should be in the most useful format, and they can be uploaded as “Source data” files linked to a main figure or table
- Include model definition files including the full list of parameters used
- Include code used for data analysis (e.g., R, MatLab)
- Avoid stating that data files are “available upon request”

Please indicate the figures or tables for which source data files have been provided:

Figure 1 has the table ‘Figure 1 – Source Data 1’, and Figure 3 has the figure ‘Figure 3 – figure supplement 1’ and table Figure 3 – Source Data 1‘.
